# Prescriber and employee utilization of a health sciences center campus outpatient pharmacy: A qualitative analysis

**DOI:** 10.1093/ajhp/zxac232

**Published:** 2022-08-18

**Authors:** Katherine S O’Neal, Emily A Oliphant, Eric J Johnson, Michael T Hines, Michael J Smith

**Affiliations:** University of Oklahoma College of Pharmacy, Oklahoma City, OK, USA; University of Oklahoma College of Pharmacy, Oklahoma City, OK, USA; University of Oklahoma College of Pharmacy, Oklahoma City, OK, USA; University of Oklahoma College of Pharmacy, Oklahoma City, OK, USA; University of Oklahoma College of Pharmacy, Oklahoma City, OK, USA

**Keywords:** focus group, health sciences center campus, on-campus pharmacy, pharmacy innovation

## Abstract

**Purpose:**

The college of pharmacy has operated pharmacies on campus for over 26 years. Employees and patients are users of the pharmacies; however, utilization across the campus has been limited. This paper describes a process, as well as results, that was used to gather input from employees on a large university health sciences center campus on pharmacy needs and related behaviors on campus pharmacy utilization.

**Methods:**

Two focus groups of staff and 4 focus groups of prescribers were conducted over 1 month. Participants were selected through purposive sampling via email within an academic health sciences center campus over a 1-month period. The sessions were moderated by one investigator using a preconstructed discussion guide and lasted 1 hour. Two additional investigators observed sessions for nonverbal communication; all sessions were audio recorded for subsequent transcription. An open-coding process was performed on verbatim transcripts using NVivo12. The investigator team then developed, refined, and grouped themes during subsequent group discussions.

**Results:**

A total of 44 participants took part in 6 focus groups. Participants included prescribers (physicians, nurses, physician assistants) and staff (nonprescribers). Two major themes identified were (1) factors related to on-campus pharmacies and (2) qualities valued in a pharmacy. There was an equal split (8% for each group) on awareness of the on-campus pharmacies. Almost 11% of participants commented on the accessibility of a pharmacy being a quality valued in a pharmacy.

**Conclusion:**

Focus groups provided insights for the administration team regarding additional value-added services that would be helpful for the campus community, as well as various approaches to increase utilization of the on-campus pharmacies. Focus group methodology is an effective approach to engage employees of a large university campus to garner new ideas to enhance existing policies or services, as well as to gather thoughts on preliminary strategic plans before implementation.

**Key Points**

- Utilization of focus groups can greatly contribute to understanding of attitudes and behaviors in any area of healthcare as well as large university health sciences centers.
- Factors related to on-campus pharmacies and qualities valued in a pharmacy were the major themes that emerged from the focus groups.
- Data produced from the focus groups generated ideas for expansion of services and offerings in our pharmacies that will better meet the needs of our campus community.

Pharmacists are highly trusted healthcare professionals and are highly accessible for patients.^1^ Patients have been documented to seek pharmacists that are patient centered and value patient-pharmacist relationships.^2,3^ Patients also perceive that competence-based attributes, specifically drug safety expertise, are a primary merit of the community pharmacist.^3^ Formalized quality measures, such as those approved by the Pharmacy Quality Alliance, are not well understood by many patients.^4^ As a result, these measures do not commonly influence patients’ choice of pharmacy.^5^ Despite nearly all Americans living within 5 miles of a community pharmacy, there is limited literature on what attributes draw patrons to a specific pharmacy and whether accessibility carries through to place of employment.^1^

A large university health sciences center (HSC) campus located centrally in a large metropolitan city houses 3 outpatient pharmacies across campus that are operated by a college of pharmacy. Other medical facilities located on the HSC campus include multiple primary care clinics, specialty clinics, an adult hospital, a children’s hospital, a cancer center, a poison control center, pharmacy benefits administration, an eye institute, and a diabetes center. The college of pharmacy also owns and operates an outpatient pharmacy located on the satellite campus of the university in another large metropolitan city. These pharmacies provide services outside of traditional dispensing, including immunizations and adherence packaging. New services, such as medication delivery for transitions of care and curbside delivery, are also offered to serve the campus community. Despite these service offerings, a survey conducted by campus administration in spring 2019 to gather employee perspectives on various campus resources and entities identified that there was low awareness and utilization of campus pharmacies. Identifying healthcare provider and campus employee perspectives, as well as desired pharmacy services, will greatly aid in enhancements to the pharmacies to better meet the needs of the HSC and surrounding community. Focus groups are an effective way to elicit perceptions from healthcare providers or consumers and can be used in any area to aid in administrative decision-making. The objective of this study was 3-fold: (1) to identify awareness of campus pharmacies and services offered; (2) to determine perspectives on what is expected from pharmacy services and what can be provided; and (3) to identify facilitators and barriers, including resource-related, attitudinal, situational, and other factors, to utilization of campus pharmacies. This study was approved by the institution’s review board committee.

## Methods

Qualitative and quantitative research methods were used to gather information from focus groups across the HSC campus. Focus group methodology is a relatively inexpensive process that allows for guided, semistructured, and in-depth conversations that seek to answer key questions while providing insightful beliefs and understanding from participants. A preconstructed guide was developed to facilitate the focus group discussions (see Appendix for a copy of the guide). Eight focus group sessions were proposed on different days between October 1 and October 31, 2019. Six focus groups consisted of nonprescribing staff employees, and 2 focus groups consisted of prescriber employees.

### Focus group guide

Grounded theory was used to develop separate guides for the focus groups targeted at prescribers vs nonprescribing staff. Grounded theory features an inductive design used to study social experiences and explain processes, as opposed to testing an existing theory.^6,7^ Consistent with this approach, study investigators developed targeted yet open-ended questions to elicit data.

The investigators operationalized the study objectives into specific, open-ended questions to elicit information from participants regarding on-campus pharmacy services. Separate guides were used for prescriber and nonprescriber staff focus groups. The questions on the guide were not piloted but were vetted internally among the college’s pharmacy and operations committee to ensure that the desired information would be captured from respondents.

### Participant selection

Purposive sampling and snowball sampling within the staff/prescriber-based groups at the HSC campus were used.^8-10^ An email describing the study and detailing information for signing up for a preidentified focus group date was disseminated to all campus employees (approximately 9,000 people) before the study start and again midway through the study. College of pharmacy employees were not eligible to participate. The focus groups were conducted at various locations within the HSC campus at either 7 am or 12 pm to accommodate the various work locations and schedules of participants. Participants emailed or called a designated study investigator to sign up for the day of their choice and eligibility (prescriber vs staff). Participants were encouraged to share the information about the study with their colleagues.

### Setting

Each focus group was moderated by one study investigator using the preconstructed interview guide.^11^ Each focus group comprised up to 10 healthcare prescribers or staff, and each session comprised either all prescribers or all nonprescriber staff. During the prescriber focus groups, each prescriber spoke on behalf of their patients as providers or themselves as employees utilizing the pharmacies. The primary difference between the 2 focus group guides was the inclusion of questions related to referring patients to the pharmacies and whether patients’ needs were being met. The sessions lasted 1 hour, and participants were provided with either a continental breakfast or lunch and a $25 gift card for their time.^12,13^ Participants could opt out of the focus groups at any time.

### Data collection

At the beginning of each focus group, the moderator introduced each member of the study team, as all team members remained for the duration of the session. Each participant then introduced themselves and described their role on campus. The discussion opened with identifying each participant’s awareness of the pharmacies on campus. The investigators recorded all focus group sessions with an audio-recording device. A third-party professional service transcribed all audio recordings. Four investigators reviewed each transcript and adjudicated any uncertainties to ensure accuracy. Two investigators also took notes during each focus group session to record nonverbal communication and to ensure proper interpretation of vocal inflections from the transcriptions.

### Data analysis

Transcripts were imported into qualitative data software (NVivo12; QSR International, Burlington, MA).^14,15^ Each investigator reviewed the transcripts for understanding using personal interpretation of the verbatim transcripts. A single investigator performed an open-coding process to generate initial codes. No formal coding template was developed. Investigators held iterative group discussions to develop, refine, and group themes.

## Results

Six focus groups were completed with a total of 19 providers and 15 staff (N = 44). All participants completed the duration of their respective focus group session. Participants learned of the study either through the email that was disseminated or through their colleagues.

**Figure 1.**
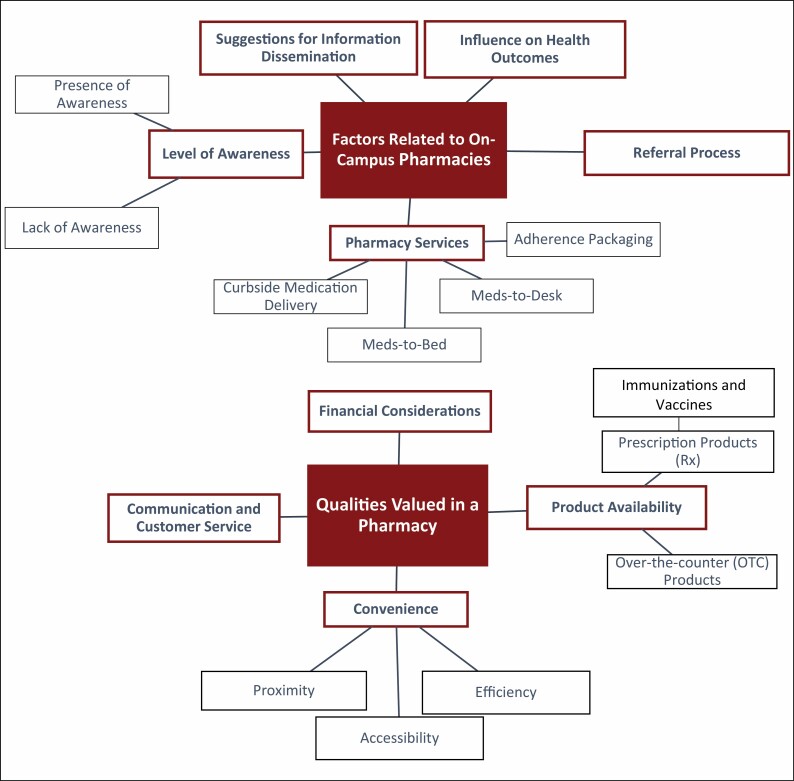
Content analysis of focus group transcripts including health sciences center employees and prescribers, October 2019.

Two major themes were identified from participants’ responses: (1) factors related to on-campus pharmacies and (2) qualities valued in a pharmacy (Table 1). Figure 1 provides an overview of the 2 themes identified with corresponding subthemes. The frequency, duration, and type of responses within each theme varied between and within each focus group session. Five subthemes emerged under factors related to on-campus pharmacies: (1) suggestions for information dissemination, (2) influence on health outcomes, (3) referral process, (4) pharmacy services, and (5) level of awareness. Four subthemes were identified under qualities valued in a pharmacy: (1) financial considerations, (2) product availability, (3) convenience, and (4) communication and customer service. Supporting quotes for a few of the subthemes under the 2 broader themes are outlined below.

**Table 1.** Nodes Identified Through Content Analysis of Focus Groups Including Health Sciences Center Employees and Prescribers, October 2019

Nodes	Percent of coded data^a^
**OUCOP on-campus pharmacies**	
Level of awareness of OU pharmacies	
Lack of awareness or familiarity	8
Presence of awareness or familiarity	8
Influence on health outcomes	0.86
Suggestions for information dissemination	5.71
Referral process	4.57
Pharmacy services	
Adherence packaging	12.86
Curbside medication delivery	2
Meds-to-bed service	4
Meds-to-desk service	2.57
**Qualities valued in a pharmacy**	
Communication and customer service	4.29
Convenience	2.86
Accessibility	10.57
Efficiency	1.71
Proximity	4.57
Financial considerations	6.57
Product availability	10
Prescriptions	3.14
Immunizations	3.14
Nonprescription products	2.57
**External (non-OUCOP) pharmacy experiences**	
Negative experiences with external pharmacies	0.86
Positive experiences with external pharmacies	0.57
Total	100

Abbreviations: OU, Oklahoma University; OUCOP, Oklahoma University College of Pharmacy.

^a^The percentage for each node represents the proportion of the total coded data that was related to each node.

### Theme 1: factors related to on-campus pharmacies

The level of awareness of the pharmacies and familiarity with the locations and hours of each varied among employees and prescribers but mostly was identified as an area for improvement (8%).

Participant 1, focus group 5 (prescriber): “*So I only know of the one that is here, so I don’t know where the other locations are*.”

Participant 2, focus group 2 (staff): “*I do not use any of them because I did not know 2 of those existed*.”

Participant 3, focus group 1 (staff): “*Yeah, that was my concern, is that I would not be able to fill a prescription on the weekends*.”

Participant 4, focus group 3 (prescriber): “*I don’t know what the typical hours are at our campus.*”

Communication and information dissemination was identified by the focus groups as a desired attribute to help raise awareness and keep employees abreast of pharmacy-related news (4.29%). Staff and prescribers suggested that information regarding the pharmacies be disseminated specifically via employee orientation and/or welcome packets and marketed via email.

Participant 5, focus group 4 (staff): “*I have never had a provider tell me there are pharmacies on campus or ask me if I have ever considered using one on campus and why I should, so with marketing, it would be kind of cool to send out an email to staff about the pharmacies and maybe why staff might want to use them over chains*.”

Participant 6, focus group 1 (staff): “*… could put in a flyer in our welcome packets for residents*”

Participant 7, focus group 6 (prescriber): “*… as part of your orientation, and (point out) here is the pharmacy*”

Participant 8, focus group 3 (prescriber): “*Do you all have a marketing plan where you’re actually marketing to faculty and staff because I bet a lot of people do not even know you’ve got three locations here.*”

Participants also displayed enthusiasm regarding recently implemented and forthcoming pharmacy services, including adherence packing (12.86%), a meds-to-desk program (2.57%), and curbside delivery of medications (2%).

Participant 9, focus group 1 (staff): “*I think if you heavily advertised that, you would have a huge amount of people that would appeal to. Because if you think about, like, how busy, like for me, for example, my day is crazy. And so, I will sit at my desk and eat lunch and so that might be like, oh, let me order my pills. They deliver them during that time, and I have them and when I get off work I do not have to go to the pharmacy or I do not have to walk across the street to go get the prescription. That would make my life so easy*.”

Participant 2, focus group 2 (staff): “*It (meds-to-desk program) sounds amazing. I think most of us would utilize it if it is available*.”

Participant 10, focus group 1 (staff): “*Oklahomans love drive-thrus. We do not like to walk.*”

### Theme 2: qualities valued in a pharmacy

Closely related to the service offerings of a meds-to-desk program and curbside delivery, the accessibility of the pharmacies, including hours of operation and parking availability, was reported by employees and prescribers of the HSC campus as a highly valued quality of pharmacy services (11%).

Participant 11, focus group 5 (prescriber): “*I think this is the only major hospital where there is no pharmacy after 7, holidays. I mean the first question people ask is like, ‘Where do I get it filled?’*”

Participant 10, focus group 1 (staff): “*Yeah, that was my concern, is that I would not be able to fill a prescription on the weekends*.”

Participant 12, focus group 5 (prescriber): “*When they need new prescriptions, it is just cumbersome to come here. It is difficult*.”

Participant 13, focus group 2 (staff): “*Parking is a big deal. It is hard to get in, hard to get out. I do not want to get a ticket.*”

Employees of the HSC campus verbalized financial considerations more frequently than other qualities outlined in the subthemes (6.57%).

Participant 14, focus group 4 (staff): “*It’s a good reminder that you can get over-the-counter stuff here for competitive prices.*”

Participant 15, focus group 1 (staff): “*What your insurance allows because sometimes your insurance will only allow you to use certain pharmacies*.”

Participant 16, focus group 4 (staff): “*Do the campus pharmacies accept discount cards?*”

Product availability of nonprescription items, prescription products, and vaccines was also addressed as a distinguishing quality of a pharmacy. Pharmacy efficiency, specifically comments regarding wait times, was mentioned infrequently relative to other topics by staff and prescribers in the focus groups. Experiences with external (not on campus) pharmacies, both positive and negative, were also referenced relative to the on-campus pharmacies but by very few people.

## Discussion

A recent organization-wide survey conducted by administration of the HSC identified low utilization of campus pharmacies among campus employees. Consumers of services in any area of healthcare can often give insight on potential improvements relating to utilization, efficiency, or safety of the care delivery processes. Focus group methodology can elicit such information and lead to effective and efficient improvements by the institution as these changes are based on the insightful perceptions of an individual or group within the organization who impact clinical and/or economic outcomes as well as strategic plans. Information garnered from focus groups can ultimately aid in administrative decision-making. Information generated from the focus group sessions reported in this paper affirmed the service expansion ideas the pharmacy operations team were currently developing along with providing new ideas for other expansion opportunities. One example of a previously identified service idea that garnered substantial excitement and lively conversation from the focus group participants and exceeded the expectations of the investigators was the meds-to-desk service. We identified this service opportunity due to access barriers such as lack of parking at the pharmacies and not having a drive-thru pharmacy. With the structural reality of the pharmacy locations in physician clinics, the pharmacy operations team discussed the idea of a delivery system that would take medications to employee work areas. Thus, the meds-to-desk delivery concept was started. It was a top discussion item by the focus group panels, which solidified plans to proceed with implementing the service. This service idea directly addresses multiple areas that panelists shared were barriers to utilization or valued qualities of pharmacies: hours of operation, convenience, and lack of a drive-thru. Several participants commented on the hours of operation for the pharmacies and weekend access. The hours were expanded in spring 2019, but the comments received during the focus group sessions highlighted that many were unaware of the updated hours and identified the need for increased marketing efforts for the pharmacies. Owing in part to the need of the hospital’s emergency department to provide access to medications quickly and in recognition of how valued drive-thru services were to the focus group participants, coupled with a general lack of parking on the HSC campus, a curbside delivery option at 2 of the pharmacy locations on the HSC campus as well as the satellite campus was implemented. This was added immediately after the focus groups concluded and was expanded to other locations in the months afterwards due to the coronavirus disease 2019 (COVID-19) pandemic to facilitate patient access to medication while limiting the number of people in the buildings and pharmacies to ensure social distancing. To address the financial concerns of employees, the pharmacy operations team also developed a discounted formulary for the pharmacies to assist both employees and patients without insurance or with financial challenges. Some of the product offerings suggested by focus group participants are also under consideration to be added as part of the nonprescription product offerings (eg, durable medical equipment).

An adherence packaging service was also implemented in March 2020 but was underutilized by patrons of the pharmacy before conducting the focus groups. The focus group discussions raised awareness that additional marketing and dissemination of information was needed as many, again, were not familiar with the service offering or even what adherence packaging entailed. It is anticipated that this service will continue to grow along with the meds-to-desk program with greater awareness through marketing efforts.

Immunizations are a service the pharmacies have offered for several years, but discussions during the focus group panels raised awareness that this, too, needed increased marketing efforts for the service offering. An interesting finding was that many prescribers were not aware that pharmacists could administer vaccines. Some expressed thoughts about the convenience they could offer their patients to have pharmacists administer vaccinations in the pharmacies located on campus.

One of the biggest opportunities identified from the focus groups was the need for increased marketing. Hence, overall marketing efforts were increased to communicate information about the pharmacies and service offerings. Examples include additional marketing materials developed to promote the upcoming meds-to-desk program. A free college of pharmacy–branded insulated lunch tote will be provided to all participants enrolling in the meds-to-desk service for the first time. Email fliers were distributed, and the college’s and university’s websites were updated to include information about the meds-to-desk program as well as expanded details on the different pharmacy locations and other service offerings. To help raise awareness of where the pharmacies are located, along with their business hours, informational brochures were created with maps identifying where the pharmacies are located, hours of operation, and contact information and were disseminated across campus clinics and academic centers. Informational televisions placed strategically throughout the various clinics and hospitals on campus were also updated to include information about the pharmacy locations and hours of operation. One of the pharmacies was remodeled, and, at the completion, an open-house event was held for all employees within the clinic building to promote awareness. Those who came to the open house got a tour of the pharmacy, met the pharmacist in charge, and received a brief overview of pharmacy services, a brochure of all pharmacy services offered, and a magnet detailing all pharmacy contact information and hours of operation. Finally, the curbside delivery service will continue, even as restrictions in place for COVID-19 begin to relax. Website and social media enhancements/functions are being reviewed to also help address barriers such as access and convenience. It is expected that utilization of the new services and continuation of recently implemented services will increase as a result of the intensive marketing efforts. There are plans to resurvey campus employees with the same questions to assess the impact of these efforts.

Readers should consider that our analysis and identified themes were based on the reviewer’s comments. The authors did not go back to the focus group participants to verify whether our themes captured their intended thoughts. However, we feel that this limitation does not compromise our findings because the leader of the focus groups would frequently repeat and verify the comments of respondents. Additionally, we had observers record the nonverbal cues of respondents as a means to increase our confidence in accurately understanding respondents’ statements and thoughts.

## Conclusion

Despite the robust conversations generated, we experienced some challenges in gathering participants for the focus groups. Although our focus group sessions generated substantial discussion among participants and the focus group leader, including several questions from participants regarding our preliminary pharmacy programs, our time was limited to 1 hour to accommodate the unique schedules of prescribers and staff working fixed shifts in clinics and departments.

Our use of focus groups to collect healthcare prescriber and campus staff perspectives will greatly aid in continued enhancement of our pharmacies’ clinical services and operations to better meet the needs of the HSC campus. Focus group methodology was found to be an effective approach to solicit consumer perceptions and can be applied to any area of healthcare or university settings. These data will aid in administrative decision-making and ultimately lead to improvements in healthcare services provided by the institution.

## Appendix

### Study Title: Campus Pharmacy Services Focus Group

#### Sample Interview Questions for Focus Group - Physicians

- Are you aware we have outpatient pharmacies on this campus? (Yes/No)
  - Do you know how many pharmacies we have?
- Can you identify where the pharmacies are?
- Are you aware we have a specialty pharmacy? (Yes/No)
- Do you use the pharmacy for personal use?
- How often do you refer patients to the campus pharmacies?
  - Why (or why not) do you send patients to the pharmacies?
- What do you value in an outpatient pharmacy?
- What do you like about the campus pharmacies?
- How is the pharmacy at meeting your patient care needs and answering questions?
- What kinds of services or product offerings would you like to see from the pharmacies?
- What is your vision for the role of the community outpatient pharmacy within the health care team?
- What are the barriers that keep you from using a particular pharmacy for personal or patient use?
  - What suggestions do you have for overcoming those barriers?
- Are you familiar with the Meds-to-Bed program?
  - Describe the services in more detail.
  - Are you comfortable with knowing how to utilize this service?
- When I say the term ‘adherence packing’, what does it mean to you?
  - Are you aware we provide the service?
  - Describe the service in more detail.
  - Are you comfortable with knowing how to utilize this service?
- Is there anything else you want others to know about the campus pharmacies?

### Study Title: Campus Pharmacy Services Focus Group

#### Sample Interview Questions for Employees

- Are you aware we have pharmacies on this campus? (Yes/No)
  - Do you know how many pharmacies we have?
- Can you identify where the pharmacies are?
- Are you aware we have a specialty pharmacy? (Yes/No)
- Do you use the pharmacy for personal use?
- What do you value in an outpatient pharmacy?
- What do you like about the campus pharmacies?
- What kinds of services or product offerings would you like to see from the pharmacies?
- What are the barriers that keep you from using a particular pharmacy?
  - What suggestions do you have for overcoming those barriers?
- When I say the term ‘meds to desk’, what does that mean to you?
  - How would a medication delivery service impact your decision to use the pharmacy?
  - What concerns would you have?
- When I say the term ‘adherence packing’, what does it mean to you?
  - Are you aware we provide the service?
